# CBT for depression: a pilot RCT comparing mobile phone vs. computer

**DOI:** 10.1186/1471-244X-13-49

**Published:** 2013-02-07

**Authors:** Sarah Watts, Anna Mackenzie, Cherian Thomas, Al Griskaitis, Louise Mewton, Alishia Williams, Gavin Andrews

**Affiliations:** 1 Clinical Research Unit for Anxiety and Depression, School of Psychiatry, University of New South Wales at St Vincent’s Hospital, 394-404 Victoria Street, Darlinghurst, NSW, Australia

**Keywords:** Cognitive behavioural therapy, Major depressive disorder, Mobile app, Internet treatment, Treatment

## Abstract

**Background:**

This paper reports the results of a pilot randomized controlled trial comparing the delivery modality (mobile phone/tablet or fixed computer) of a cognitive behavioural therapy intervention for the treatment of depression. The aim was to establish whether a previously validated computerized program (The Sadness Program) remained efficacious when delivered via a mobile application.

**Method:**

35 participants were recruited with Major Depression (80% female) and randomly allocated to access the program using a mobile app (on either a mobile phone or iPad) or a computer. Participants completed 6 lessons, weekly homework assignments, and received weekly email contact from a clinical psychologist or psychiatrist until completion of lesson 2. After lesson 2 email contact was only provided in response to participant request, or in response to a deterioration in psychological distress scores. The primary outcome measure was the Patient Health Questionnaire 9 (PHQ-9). Of the 35 participants recruited, 68.6% completed 6 lessons and 65.7% completed the 3-months follow up. Attrition was handled using mixed-model repeated-measures ANOVA.

**Results:**

Both the Mobile and Computer Groups were associated with statistically significantly benefits in the PHQ-9 at post-test. At 3 months follow up, the reduction seen for both groups remained significant.

**Conclusions:**

These results provide evidence to indicate that delivering a CBT program using a mobile application, can result in clinically significant improvements in outcomes for patients with depression.

**Trial registration:**

Australian New Zealand Clinical Trials Registry ACTRN 12611001257954

## Background

Depression is a commonly occurring, disabling mental disorder [1-3]. Worldwide it is currently the fourth leading cause of disability and is expected to become the second leading cause of disease burden by the year 2020 [4]. Cognitive behavior therapy (CBT) has been shown to be effective in the treatment of depression [5,6]. However, a number of barriers prevent patients from accessing treatment. For example, three–quarters of people in the UK with depression received no treatment, with cost being the major barrier [7,8]. Treatments that are more affordable and accessible are necessary.

CBT via the internet (iCBT) has been shown to be as effective as face to face treatment for Major Depressive Disorder (MDD) and more cost effective [9,10]. Johanssen and Andersson (2012) reviewed 25 controlled trials of iCBT for MDD [11]. Effect size (ES) superiority of the intervention over the control group ranged between 0.1 and 1.2, but there were six studies in which the effect size superiority was greater than 0.85 (mean = 1.0, NNT=2). The intervention in one of these [12,13](ES=1.2) was the basis of the present work.

Delivery of CBT using the internet has increased the options available for patients with depression to access evidenced based effective treatment. For some, using a fixed computer may mean little or no privacy, or it may simply be inconvenient. Moreover, a patient may want to review the treatment lesson material in context, for example, re-reading material on a computer just prior to entering a challenging situation may not be feasible. For others, access to the internet may be unreliable and this may not be a useful alternative. It is for these reasons, that alternative modes of treatment delivery should be considered. Mobile applications (apps) offer a viable, cost effective and highly accessible solution. Recently mobile apps have been utilized to deliver health treatments [14,15] and whilst a plethora of apps are available, evidence based, evaluated apps remain sparse. With almost 6 billion mobile phone subscriptions worldwide in 2011, it is anticipated that this mode of health delivery will rise [16]. To our knowledge, there are no published RCTs using a mobile app to deliver treatment for MDD. Using a mobile app may enable greater choice in preferred treatment options, increased convenience, greater accessibility and enhanced privacy.

Bang and colleagues (2007) have described the advantages of using a mobile phone over other delivery methods for some of the elements of CBT, explaining a mobile phone has the functionality to record, scale and label anxiety-provoking situations when and where the need arises [17]. These advantages were evidenced in a recent study that showed people’s everyday mood, stresses, responses and general functioning, can be helpfully communicated to primary care practitioners by tracking and capturing data in context [18]. Furthermore, participants from this study reported this method of data collection as convenient and acceptable. This positive sentiment was echoed in a study exploring community attitudes towards the use of mobile phones for mental health monitoring and self management. Proudfoot and colleagues found attitudes were positive towards using a mobile phone for mental health monitoring, however, participants identified the importance of privacy, security provisions and an easy to use program [19].

On this basis, we revised our previously evaluated 6-lesson clinician assisted treatment program for depression (The Sadness Program) [11,12] into a mobile app version, complete with security settings. The name of the program was changed from The Sadness Program to The Get Happy Program to convey a sense of optimism and empowerment. In order to demonstrate the efficacy of the program using a mobile app, we decided to compare the delivery mode of the same program between a mobile vs. computer group. It was hypothesized that all participants would show significant improvement on measures of depression, a reduction of psychological distress, and participants would find the treatment modes equally acceptable. Given the transportable nature and use of mobile phones, we thought it would be important to understand the type of environment utilized, and if, any, environmental factors, such as noise, may have an impact on the efficacy of the program for mobile users. Thus, based on the Experience Sampling Method [20], three items were constructed to identify the location of where the participant was completing the lesson, how distracting the level of noise was, and the self rated ability of the participant to concentrate in this environment. Adherence towards homework was also measured using self reported effort and amount of homework completed.

## Method

### Design

A CONSORT-2010 compliant, registered RCT compared two modes of delivery, a mobile versus computer group. Both groups were followed through 3-months follow-up.

### Participants, randomisation, and recruitment

**Figure 1:**
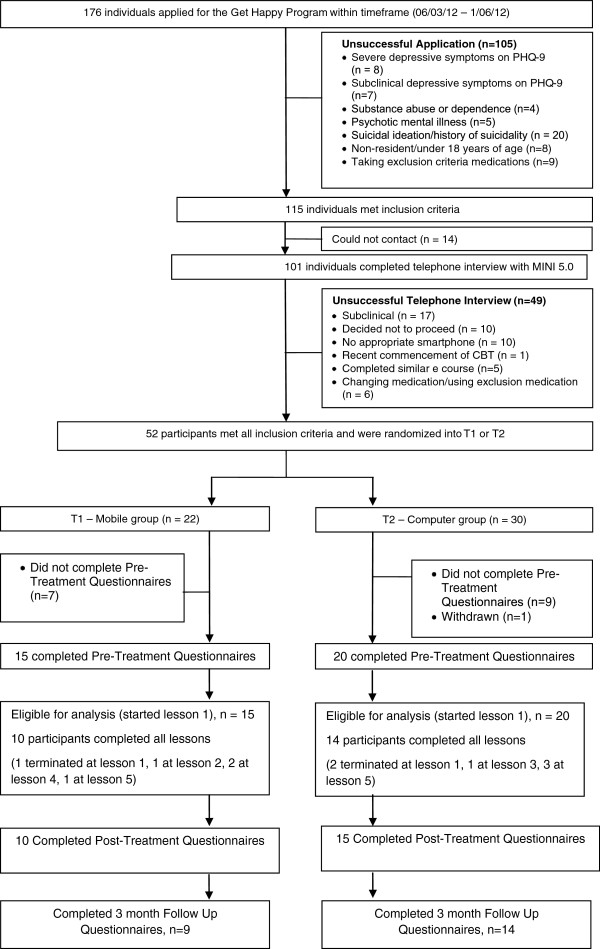
Flow of participants through trial.

Applicants applied to http://www.virtualclinic.org.au after reading details about the study. Details of the applicant and participant flow are in Figure 1. During recruitment between March and May 2012, 176 individuals applied for this program and 52 applicants met the inclusion criteria: (i) aged over 18, (ii) self identified as suffering from mild or moderate depression, and have PHQ-9 scores and results of telephone diagnostic interview (MINI) consistent with this, (iii) prepared to provide name, phone number and address, and (iv) to provide written informed consent, (v) had access to a mobile phone or iPad, and a computer with a printer and, (iv) had previous experience with downloading a mobile app. Applicants were excluded if they: (i) had psychosis, bipolar disorder, substance abuse or dependence, (ii) severe depression (with a PHQ-9 score greater than 24) and (iii) or current suicidality, as assessed by Question 9 on the PHQ-9: “how frequently over the last two weeks have you been bothered by thoughts that you would be better off dead or of hurting yourself in some way?” If the applicant answered 2 (= more than half the days), with a history of previous suicide attempts or answered 3 (= nearly every day), with or without previous suicide attempts, they were excluded, and contacted by the study clinicians (CT, AG) and advised as to an appropriate course of action.

115 applicants met selection criteria and of these 101 were able to be contacted by phone. The content of the call included a review of the study and confirmation that the applicant had read and understood the information and consent form. Following this, the Mini International Neuropsychiatric Interview Version 5.0.0 (MINI) [21] was used to confirm the applicant met DSM-IV criteria for a Major Depressive Disorder (MDD). 52 applicants met all inclusion criteria and were randomised via a true randomisation process (http://www.random.org) generated by a team member not involved in the study, to either the Mobile Group (*n*=22) or Computer Group (*n*=30). However, at baseline we had 15 participants in the Mobile Group and 20 in the Computer Group, due to participants not starting the first lesson. Concealment of allocation was maintained until the applicant met all inclusion criteria and an offer of participation made. The unsuccessful applicants (*n*=49) were advised about more appropriate treatment options. The study was approved by the Human Research Ethics Committee (HREC) of St Vincent’s Hospital (Sydney, Australia) and the trial was registered as ACTRN 12611001257954.

### Diagnostic measure

#### Mini international neuropsychiatric interview version 5.0.0 (MINI)

The MINI is a brief diagnostic interview developed to determine the presence of a current and life-time Axis-1 disorder using DSM-IV diagnostic criteria. It has excellent inter-rater reliability (k=.88-1.00) and adequate concurrent validity with the Composite International Diagnostic Interview [22].

### Description of treatment

**Figure 2:**
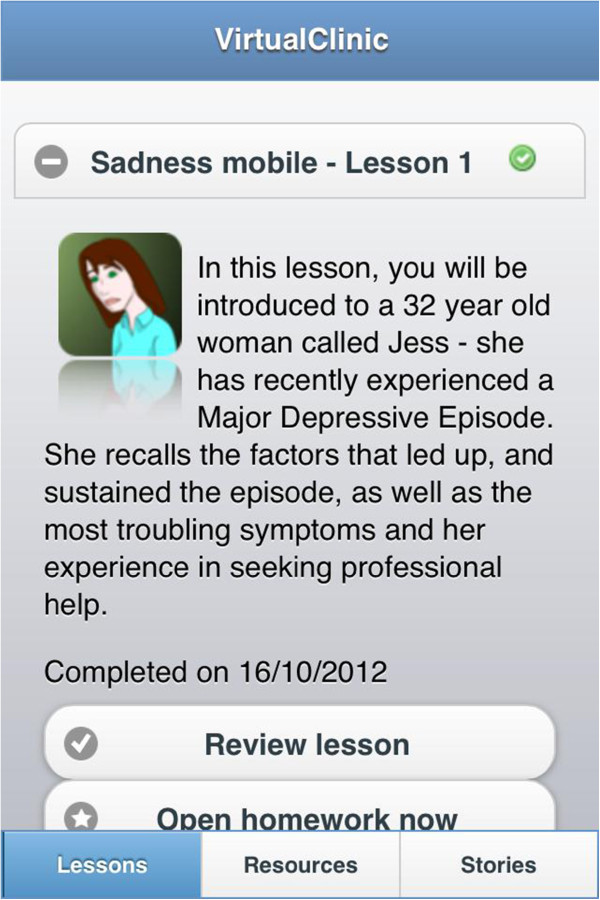
Screenshot of mobile program outlining available options for mobile participants to review lesson, open homework, access resources or read stories.

**Figure 3:**
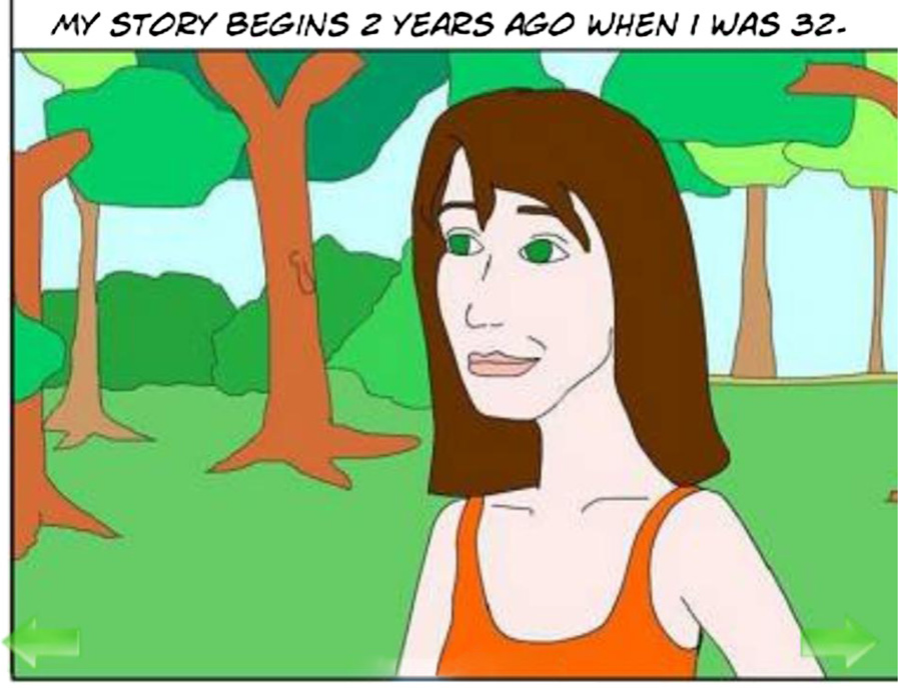
Screenshot of mobile version of program.

The Get Happy Program was based on the principles of CBT and is a version of the previously evaluated Sadness Program [11,12]. The program consisted of 6 lessons conducted over an 8 week period. The lessons read like a comic book and participants follow the story of Jess, a comic character that has depression, and through her story learn how she comes to manage her symptoms, and participants can then apply these principles to their own life. The size of the font was adapted for the mobile version and some minor revisions were made to the content of the program. On completion of each lesson, participants were assigned and encouraged to carry out the relevant homework activities and review the lesson. Additional resources, such as information on assertiveness skills and sleep hygiene, and stories from previous participant’s experiences were also available Figure 2, Figure 3.

### Clinician contact

Participants received emails/or phone calls from a clinician, until completion of Lesson 2 because our previous research study has found that additional clinician support does not add any further benefit to participants [13]. Following this, the only clinical contact was when the participant initiated contact, or when a clinician instigated contact due to a deterioration in the K-10 score [23]. The K-10 is a measure of psychological distress and was completed prior to beginning each lesson.

### Outcome measures

*The Patient Health Questionnaire-9* (PHQ-9) [24] is a brief 9-item self-report scale that measures each of the DSM-IV criteria for MDD with scores ranging from 0 to 27. Participants rate the frequency of symptoms over the last fortnight on a scale ranging from 0 (not at all) to 3 (nearly every day), where 1 = several days, 2 = more than half of the days. A PHQ-9 score of ≥10 is used as a clinical cut-off for probable DSM-IV diagnosis of MDD [25]. The PHQ-9 has been shown to have good sensitivity and specificity [26] and excellent reliability and validity [24].

*The Kessler 10-item Psychological Distress scale* (K-10) [23] is a measure of non-specific psychological distress over the past 14 days. Scores can range from 10 to 50, with higher scores indicating higher distress. The K-10 has good psychometric properties and can discriminate between cases and non-cases of DSM-IV affective disorders [27].

*Beck’s Depression Inventory Second Edition (BDI-II)*[28] consists of 21 items and is a measure of the presence and severity of a MDD based on the DSM-IV diagnostic criteria for depression. Scores range from 0 to 63 with higher scores indicating a greater severity of depression. The BDI-II possesses excellent internal consistency [28].

*Credibility/Expectancy Questionnaire (CEQ)*[29] is a scale for measuring treatment credibility and expectancy of improvement. At post treatment, 2 items based on the CEQ were asked including, how satisfied were you with the skills that this program has taught you to manage your depression from 1 (not at all satisfied) to 9 (very satisfied); how confident would you be in recommending this treatment to a friend who experiences similar problems from 1 (not at all confident) to 9 (very confident).

*Sheehan Disability Scale (SDS)*[30] is a measurement of functional disability and impairment due to psychiatric symptoms and is self rated. The 2 items related to productivity were used for this study. The first item used to measure absenteeism asked participants how many days in the last week their symptoms caused them to miss studies or work, or leave them unable to do their normal daily responsibilities. The second item used to measure presenteeism asked participants on how many days in the last week did they feel so impaired by their symptoms, that even though they studied, went to work or worked at home, their productivity was reduced.

#### Environment rating scale (ERS)

Three items were constructed to identify the location of where Mobile Group participants were completing the lessons with options including: at home, at work, on a train or a bus, at a park or the beach, a café, or other. The Mobile Group participants were also asked to rate how distracting the level of noise was from 1 (no distracting noise) to 4 (extremely distracting noise). Lastly, the Mobile Group participants were asked to rate their ability to concentrate in this environment from 1 (poor) to 4 (excellent) (adapted from the Experience Sampling Method, [20]).

#### Homework rating scale (HRS)

Two items were constructed to measure the amount of effort and homework completion. The participant was first asked how much effort they had put into the homework from 0 (no effort) to 4 (complete effort); and how much of the assigned homework they had finished from 0 (none) to 4 (all).

### Statistical analysis

For the PHQ-9, K-10 and BDI-II analyses were conducted using linear mixed-model repeated measures (MMRM) ANOVA with measurement occasion as a within-group factor and intervention as a between-groups factor. These analyses were conducted using the MIXED procedure in SPSS Version 19 with an identity covariance matrix. For the SDS generalised estimating equations (GEE) were used to evaluate reductions in absenteeism and presenteeism across time. GEE is a semi-parametric method used to implement marginal models when the outcome variable is not normally distributed. The GENLIN procedure with a repeated statement was implemented using SPSS Version 19. A poisson distribution with a log link function was specified. An unstructured covariance structure was used to model the within-subject dependencies. Initial models only included measurement occasion, study group and their interaction as fixed effects. For each outcome measure, the two homework questions were included separately in subsequent models to investigate whether any of the outcomes differed based on self-reported homework effort or homework completion.

### Outcome measurement

All participants completed the questionnaire outcome measures (BDI-II, PHQ-9, K-10, CEQ, ERS and SDS) at 1-week post treatment and at 3-months follow up.

## Results

### Baseline

The mean age of participants was 41 years (*SD*= 12.38, range =18 - 63) and 28/35 were female (80% of the sample). Participants reported on average, moderate depression levels using the PHQ-9, severe levels of depression on the BDI –II, and severe levels of psychological distress using the K-10. Participants reported on average 2.14 days of work lost (*SD*= 2.19) and 4.40 days of work that were underproductive (*SD*= 2.15) due to the presence of his/her psychiatric symptoms on the SDS (see Table 1).

**Table 1:** Baseline, mid, post-treatment estimated means, standard deviations, MMRM ANOVA, and effect sizes for outcome measures

	**Pre-treatment**	**Mid-treatment**	**Post-treatment**	**Follow up**	**Statistic**				**Effect Size**	
Measure	M (SD) (*n*=35)	M (SD) (*n*=30)	M (SD) (*n*=25)	M (SD) (*n*=23)	Pre to post within group (measurement occasion)	Pre to post between group (time by study group interaction)	Pre to follow up within group (measurement occasion)	Pre to follow up between group (time by study group interaction)	Within-subjects pre- post- treatment comparison for treatment group (95% confidence intervals)	Between group post treatment comparison (95% confidence intervals)
**PHQ-9** Mobile	14.65 (1.37)	6.45 (1.51)	6.55 (1.51)	5.28 (1.63)	*F* [2, 51.97] = 33.22, *P*=<.001	*F* [2, 51.97] = 1.09, *P*=.34	*F* [3, 73.6] = 28.4, *P*=<.001	*F* [3, 73.6] = .875, *P*=.458	1.41 (.55-2.26)	−0.47 (−0.47- 0.20)
**PHQ-9** Computer	14.20 (1.62)	8.98 (1.24)	7.21 (1.26)	7.18 (1.32)					.92 (.19-1.64)	
**BDI-II** Mobile	33.46 (2.95)		12.53 (3.26)	11.66 (3.47)	*F* [1, 25.55] = 86.02, *P*=.<.001	*F* [1, 25.55] = .41, *P*=.52	*F* [2, 47.09] = 60.1, *P*=.<.001	*F* [2, 47.0] = 1.7, *P*=.19	1.79 (0.92-2.65)	−0.37 (−1.05- 0.29)
**BDI-II** Computer	30.90 (2.55)		13.68 (2.79)	16.75 (2.85)					1.88 (1.14-2.62)	
**K-10** Mobile	30.60 (2.06)	22.44 (2.21)	20.03 (2.21)	19.74 (2.31)	*F* [6, 153.9] = 24.3,*P*=<.001)	*F* [6, 153.9] = .359, *P*=.90)	*F* [7, 1734.5] = 28.4, *P*=<.001)	*F* [7,174.91] = .370, *P*=.919)	1.05 (.20-1.89)	0.03 (−0.63-0.70)
**K-10** Computer	30.15 (1.78)	24.12 (1.86)	19.95 (1.88)	19.55 (1.93)					1.22 (.52-1.92)	
**SDS** days lost Mobile	2.20 (.62)		.74 (.42)							
**SDS** days lost Computer	2.10 (.44)		1.15 (.37)							
**SDS** days underproductive Mobile	4.13 (.54)		2.23 (.71)							
**SDS** days underproductive Computer	4.25 (.47)		1.89 (.53)							

### Baseline between-group comparisons

Independent samples t-tests compared the two groups on baseline demographic characteristics and pre-treatment symptom questionnaires. There were no differences between the groups on age, the BDI-II, PHQ-9, nor K-10.

### Adherence results

8.6% (3/35) completed only the first lesson, 2.9% (1/35) completed two lessons, 2.9% (1/35) completed 3 lessons, 5.7% (2/35) completed 4 lessons, 11.4% (4/35) completed 5 lessons and 68.6% (24/35) of participants completed all six lessons. Refer to Figure 1. When comparing the Mobile Group with the Computer Group on adherence, there were no significant differences (*t* (33) = −.242, *P* >.05).

### Disorder-specific and generic outcome measures

**Figure 4:**
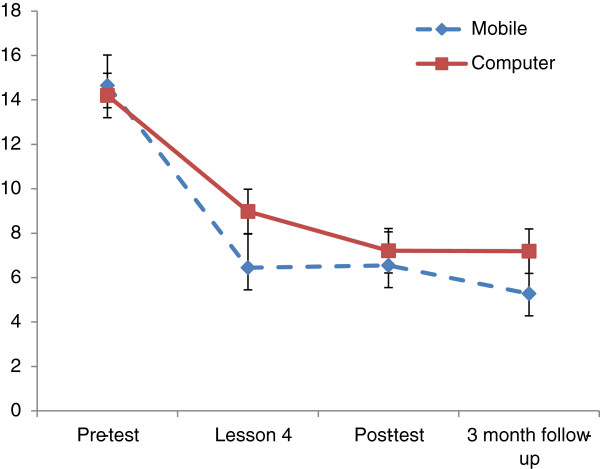
Estimated marginal means for PHQ-9 scores estimated under occasion x intervention model.

Figure 4 displays mean PHQ-9 scores on each measurement occasion as a function of the two conditions.

Statistical tests showed that the benefits of the intervention remained significant in both groups at follow up when using the PHQ-9 (*F* [3, 73.6] = 28.4, *P*=<.001), the BDI-II (*F* [2, 47.09] = 60.1, <.001) and the K-10 (*F* [7, 1734.5] = 28.4, *P*=<.001).

Estimated marginal means for PHQ-9 scores estimated under occasion x intervention model).

Results from the MMRM (ANOVA) indicated that the interaction between experimental group and time was not statistically significant when comparing PHQ-9 scores (*F* [3, 73.6] = .875, *P*=.458), BDI-II scores (*F* [2, 47.0] = 1.7, *P*=.19), or K-10 scores (*F* [7, 174.91] = .370, *P*=.919).

In order to investigate differences between each occasion of measurement, a series of pair wise comparisons were conducted. For the PHQ-9 and K-10 the change from baseline to mid-point, post-test, and 3-month follow up was investigated. Significant reductions were found between baseline and all other time points for both the Mobile Group and Computer Group. This pattern was repeated using the BDI-II, when investigating changes from baseline to post-test and 3-months follow up.

### Homework completion and effort

The aggregated mean score for homework effort for the Mobile Group was 11.25 (range, 6–17) and 14.46 (range 9–20) for the Computer Group. An aggregated mean score for homework completion for the Mobile Group was 11 (range, 8–16) and 12.4 (range 6–18) for the Computer Group. Results from the MMRM (ANOVA) indicated that there were no significant differences in homework completion or homework effort between the groups on the PHQ-9, the BDI-II, or the K-10.

### Effect sizes

Large (>.8) within-group effect sizes were found on the BDI-II, PHQ-9 and K-10 measures from pre-treatment to post-treatment. Effect sizes for these measures are included in Table 1.

### Productivity outcome measures

Statistical tests using the SDS showed a significant reduction in the number of days lost (absenteeism) (Wald Chi-Square =10.31, *P* = <.05) and in the number of days underproductive (presenteeism) from pre-treatment to post-treatment (Wald Chi-Square =12.33, *P* = .001). Further results indicated that the interaction between experimental group and time was not statistically significant when comparing the SDS on absenteeism (Wald Chi-Square =.86, *P* = .35) and presenteeism (Wald Chi-Square =.22, *P* = .63).

### Environment, distraction, and concentration measure- mobile group

Descriptive data on the environment, level of distraction and ability to concentrate, was collected for participants in the Mobile Group. Over the six lessons, 66.7-92.9% of participants completed the lessons in their home, 46.7-60% of participants completed these lessons when there was slight distraction, and 30.8-53.3% endorsed an ‘okay’ ability to concentrate. Please refer to Table 2.

**Table 2:** Environment, levels of distraction, and levels of concentration for the Mobile Group

	**Lesson 1**	**Lesson 2**	**Lesson 3**	**Lesson 4**	**Lesson 5**	**Lesson 6**
**Environment**						
Home	13/ 15 (86.7%)	13/14 (92.9%)	9/13 (69.2%)	9/12 (75%)	9/11 (81.8%)	6/9 (66.7%)
Work	0	0	2/13 (13.3%)	3/12 (25%)	1/11 (9.1%)	0
Train/bus	0	0	1/13 (6.7%)	0	0	2/9 (22.2%)
Other	2/15 (13.3%)	1/14 (7.1%)	1/13 (6.7%)	0	1/11 (9.1%)	1 (11.1%)
Missing data	0	1	2	3	4	6
**Level of distraction**						
No distraction	5/15 (33.3%)	6/14 (42.9%)	5/13 (38.5%)	5/12 (41.7%)	4/11 (36.4%)	4/10 (40%)
Slightly distraction)	7/15 (46.7%)	7/14 (50%)	7/13 (53.8)	5/12 (41.7%)	6/11 (54.5%)	6/10 (60%)
Moderately distracting)	3/15 (20%)	1/14 (7.1%)	0	2/12 (16.6%)	1/11 (9.1%)	0
Extremely distracting	0	0	1/13 (7.7)	0	0	0
Missing data	0	1	2	3	4	5
**Ability to concentrate**						
Poor	0	0	0	0	0	0
Okay	8/15 (53.3%)	6/14 (42.9%)	4/13 (30.8%)	6/12 (50%)	5/11 (45.4%)	2/10 (20%)
Good	4/15 (26.7%)	7/14 (50%)	5/13 (38.4%)	2/12 (16.6%)	4/11 (36.4%)	7/10 (70%)
Excellent	3/15 (20%)	1/14 (7.1)%	4/13 (30.8%)	4/12 (33.4%)	2/11 (18.2%)	1/10 (10%)
Missing data	0	1	2	3	4	5

### Clinical significance

At post-treatment, only 16/35 (45%) met criteria for depression on the PHQ-9 (see Table 3 for results).

**Table 3:** PHQ-9 scores according to clinical cut-off ranges at pre- and post- treatment

	**Pre treatment**		**Post treatment**	
PHQ-9 Severity Status	Mobile (*n*=14)	Computer (*n*=20)	Mobile (*n*= 11)	Computer (*n*= 16)
None (0–9)	2/14 (14%)	2/20 (10%)	8/11 (73%)	10/16 (62%)
Mild (10–14)	6/14 (43%)	11/20 (55%)	2/11 (18%)	4/16 (25%)
Moderate (15–19)	4/14 (29%)	4/20 (20%)	1/11 (9%)	1/16 (6.5%)
Severe (20+)	2/14 (14%)	3/20 (15%)	0	1/16 (6.5%)
Missing Data	1	0	4	4
Meet criteria for MDD	12/14 (89%)	18/20 (90%)	3/11 (27%)	6/16 (37.5%)

*NB.* Due to the time lag between recruitment and upon commencement of the program four participants did not meet criteria for MDD.

### Time spent per participant for both the mobile and computer groups

The mean clinician time spent per participant was 4.1 minutes (*SD*= 4.63) and the mean technician time spent per participant was 6.4 minutes (*SD*= 5.38). Each time a clinician or technician had contact with a participant the amount of time was recorded to monitor the time required to deliver the program.

### Participant satisfaction

Upon completion of the program 54% of Mobile Group and 64% of the Computer Group were very satisfied with the program; with the remaining participants endorsing ‘somewhat satisfied’. 64% of the Mobile Group and 64% of the Computer Group would be very confident in recommending this treatment to a friend; and the remainder endorsed ‘somewhat confident’.

## Discussion

These results indicate that reductions in PHQ-9, the BDI-II and K-10 pre- to post-intervention and pre to follow up, were significant, regardless of experimental group. This provides preliminary support for the efficacy of a CBT program delivered using a mobile phone. The results, including the effect size, shown in this study are commensurate with our previous RCT’s [12,13], indicating that using a mobile phone to offer this program shows similar promise.

There were a number of limitations to this study. Firstly, the small sample size necessitates replication, in order to reproduce the benefits identified in this study of delivering a CBT program via a mobile phone or computer. Other possible limitations include the self-selecting nature of the sample. Those applying to complete treatment programs using technology must be motivated; however, this does not make the results of this study invalid. A further limitation is the absence of a control group. A control group would have provided the additional advantage of ensuring the effects observed were able to be explained by the treatment. However, due to the small sample size of this study, a control group was not included. Lastly, this study did not collect data on the environment, level of concentration and distractibility in the Computer Group. Future research could explore and compare this data to inform recommendations as to the best placed environment for patients to complete the program in order to obtain the most optimal results.

The results of this pilot study indicate the usefulness of replicating this study in the future research with a larger sample size and control group. Minor additions to utilise the functionality available on a mobile phone/tablet are planned, for example, including the ability for participants to set automated reminders in their calendar, and complete the homework on their phone/tablet. Data on the time of day and amount of usage for the mobile users is also intended to be collected to understand if the increased proximity of the mobile, does lead to increased use, and greater benefits over time. Very little is currently known about the possible benefits of offering patient’s treatment programs using a mobile app on their mobile phone, however, the ubiquity of mobile apps, the growing community uptake and interest in this form of technology, and low cost associated with access, indicates that this area of research necessitates attention.

In summary, depression is common and costly. Affordable, accessible and innovative interventions should be developed, evaluated, and made available to improve the lives of those affected by this disorder. Mobile based interventions can be easily implemented and can be made widely available to the community at large. The patients and their families affected by depression deserve the opportunity to recover, and readily accessible, evidenced based treatment via a mobile phone/tablet offers hope, and an opportunity to transform current clinical practice.

## Competing interest

The authors have declared that no competing interests exist.

## Authors’ contributors

SW drafted the initial manuscript, prepared and cleaned the data and conducted initial data analysis. SW, AM, CT, AL, AW and GA conceived and performed the experiment. LM conducted further statistical analysis. GA supervised and took responsibility for the data. All authors read and approved the final manuscript.

## Pre-publication history

The pre-publication history for this paper can be accessed here:

http://www.biomedcentral.com/1471-244X/13/49/prepub
